# Cox Model Setup May Lead to Erroneous Conclusions

**DOI:** 10.1289/ehp.1205573

**Published:** 2012-10-01

**Authors:** Peter Morfeld

**Affiliations:** Institute for Occupational Epidemiology and Risk Assessment, Evonik Industries, Essen, Germany, E-mail: peter.morfeld@evonik.com

I read with interest the extensive study on lung cancer and elemental carbon exposure in trucking industry workers (Garshick et al. 2012). I believe that the Cox model setup the authors used generated potentially distorted results.

Garshick et al. (2012) used proportional hazard regression to estimate associations between lung cancer mortality and elemental carbon (EC). They adjusted for age and lung cancer secular trends by generating risk sets using attained age in 1-year increments as the timeline; they also included an ordinal variable for calendar year (1985–2000) in all models. It follows that the models were adjusted for year of birth (because year of birth = calendar year – attained age in years).

In addition, Garshick et al. (2012) noted that

To meet the assumptions of proportional hazards, we assigned separate baseline hazards based on decade of hire (< 1960, 1960–1969, 1970–1979, ≥ 1980) and age in 1985 (40 to < 50, 50 to < 60, 60 to < 70, ≥ 70 years). For example, the baseline hazard for a person 40 years of age in 1985 (born in 1945) who began work in 1975 was the same as that for all workers in their 40s in 1985 who were also hired in the 1970s . . . .

As the authors correctly concluded, this stratification of baseline hazards adjusts for decade of birth.

Because Garshick et al. (2012) used both approaches together in one analytical setup, they adjusted twice for year of birth within the same model (although with different coarseness). Thus, the results may be distorted (e.g., probable overadjustment, potential collinearity).

Furthermore, the authors

conducted sensitivity analyses with and without total years of employment as a time-dependent covariate (modeled as either continuous or in quartiles) to assess its effect as a potential confounder.

Adjusting cumulative exposure by duration of employment time-dependently reduces cumulative exposure to an estimate of long-term average concentration. However, models that directly estimate the effect of average exposure appear to be preferable (also reported on by the authors). An adjustment of cumulative exposure by total duration of employment should not be confused with an approach adjusting for the healthy worker survivor bias (Rothman et al. 2008). Thus, the sensitivity analyses Garshick et al. (2012) used to adjust cumulative exposures by duration of employment did not produce the correct effect estimates for cumulative exposure.

In summary, the Cox analyses appear to be misspecified and results cannot be interpreted in a straightforward way.
